# Personalizing progressive changes to brain structure in Alzheimer's disease using normative modeling

**DOI:** 10.1002/alz.14174

**Published:** 2024-09-05

**Authors:** Serena Verdi, Saige Rutherford, Charlotte Fraza, Duygu Tosun, Andre Altmann, Lars Lau Raket, Jonathan M. Schott, Andre F. Marquand, James H. Cole, for the Alzheimer's Disease Neuroimaging Initiative

**Affiliations:** ^1^ Centre for Medical Image Computing University College London London UK; ^2^ Dementia Research Centre UCL Queen Square Institute of Neurology London UK; ^3^ Donders Centre for Cognitive Neuroimaging Donders Institute for Brain Cognition and Behaviour Radboud University Nijmegen the Netherlands; ^4^ Department of Cognitive Neuroscience Radboud University Medical Centre Nijmegen the Netherlands; ^5^ Department of Radiology and Biomedical Imaging University of California San Francisco San Francisco California USA; ^6^ Department of Clinical Sciences Lund University Malmö Sweden

**Keywords:** Alzheimer's disease, disease progression, longitudinal serial magnetic resonance imaging, mild cognitive impairment, neuroimaging, normative modeling

## Abstract

**INTRODUCTION:**

Neuroanatomical normative modeling captures individual variability in Alzheimer's disease (AD). Here we used normative modeling to track individuals’ disease progression in people with mild cognitive impairment (MCI) and patients with AD.

**METHODS:**

Cortical and subcortical normative models were generated using healthy controls (*n* ≈ 58k). These models were used to calculate regional *z* scores in 3233 T1‐weighted magnetic resonance imaging time‐series scans from 1181 participants. Regions with *z* scores < –1.96 were classified as outliers mapped on the brain and summarized by total outlier count (tOC).

**RESULTS:**

tOC increased in AD and in people with MCI who converted to AD and also correlated with multiple non‐imaging markers. Moreover, a higher annual rate of change in tOC increased the risk of progression from MCI to AD. Brain outlier maps identified the hippocampus as having the highest rate of change.

**DISCUSSION:**

Individual patients’ atrophy rates can be tracked by using regional outlier maps and tOC.

**Highlights:**

- Neuroanatomical normative modeling was applied to serial Alzheimer's disease (AD) magnetic resonance imaging (MRI) data for the first time.
- Deviation from the norm (outliers) of cortical thickness or brain volume was computed in 3233 scans.
- The number of brain‐structure outliers increased over time in people with AD.
- Patterns of change in outliers varied markedly between individual patients with AD.
- People with mild cognitive impairment whose outliers increased over time had a higher risk of progression from AD.

## 1 BACKGROUND

The pathologies underlying Alzheimer's disease (AD) interact with an individual's distinct genetics, environmental exposures, and comorbidities, leading to idiosyncratic patterns of brain atrophy that change dynamically as the disease progresses.^1^, ^2^, ^3^, ^4^ Heterogeneity in atrophy is likely to impact individual differences between patients with AD, including timing and focality of initial symptoms and the pattern and progression of symptoms.^3^ This heterogeneity creates challenges in the clinic (e.g., when predicting prognosis and planning care), and complicates research recruitment and clinical trial design.^5^, ^6^, ^7^, ^8^, ^9^, ^10^ Therefore, there is a need to quantify disease heterogeneity at the individual level.^11^

Neuroimaging provides insights into brain structure in vivo, and has long been used to study AD; however, most studies focus on group‐average or subtype effects and overlook the individual variability between patients.^12^, ^13^ Neuroanatomical normative modeling, an emerging technique that captures individual‐level variability in the brain, has been developed to overcome reliance on group averages.^14^, ^15^ Based on the well‐established normative modeling concept, for example, height and weight growth charts for children,^16^ the neuroanatomical version builds separate normative models per brain region, based on a large independent reference dataset. An individual's brain scan can then be compared to this reference database to determine whether their brain volume or cortical thickness is lesser or greater than expected for someone of their age and sex. This deviation from normality can be quantified using *z* scores, from which brain‐wide *z* score maps can be generated, providing a unique fingerprint of an individual's brain health.^3^ Neuroanatomical normative modeling has the potential to detect specific patterns of brain changes in individual patients with AD, paving the way for personalized health care and precision medicine approaches.

Using neuroimaging data from the Alzheimer's Disease Neuroimaging Initiative (ADNI) we have previously shown the heterogeneous nature of cortical thinning patterns between individuals with AD.^17^ Here, the individualized brain‐wide *z* score maps revealed heterogenous atrophy patterns in the AD group; the extent of cortical thinning (i.e., in terms of deviation from the norm) in mild cognitive impairment (MCI) was predictive of conversion to AD and was also related to cognitive function, amyloid beta, phosphorylated tau, and apolipoprotein E (*APOE*) genotype. In a further study, individualized atrophy patterns were shown to relate to disease severity, presenting phenotypes and comorbidities in amyloid‐positive AD patients in a “real‐world” memory clinic setting.^18^ Insights from these studies have so far been based on cross‐sectional neuroimaging data. Understanding how patterns of atrophy change over time may provide insights into causal mechanisms, and aid in prognostication on an individual patient basis and when monitoring disease progression for clinical trials.

Here, we apply neuroanatomical normative modeling to quantify regional changes in brain structure as AD progresses, using serial magnetic resonance imaging (MRI) data. The neuroanatomical normative modeling can also be optimized by including controls scanned at the same site of the research cohort (adaptive learning) to reduce the impact of scanner effects.^14^ We assess whether markers of regional brain atrophy derived from normative modeling (1) can track disease trajectories in people with MCI and patients with AD; (2) can be used to predict progression from MCI to AD; and (3) are related to other common imaging and non‐imaging AD markers.

## 2 METHODS

### 2.1 Participants and research dataset

**FIGURE 1 alz14174-fig-0001:**
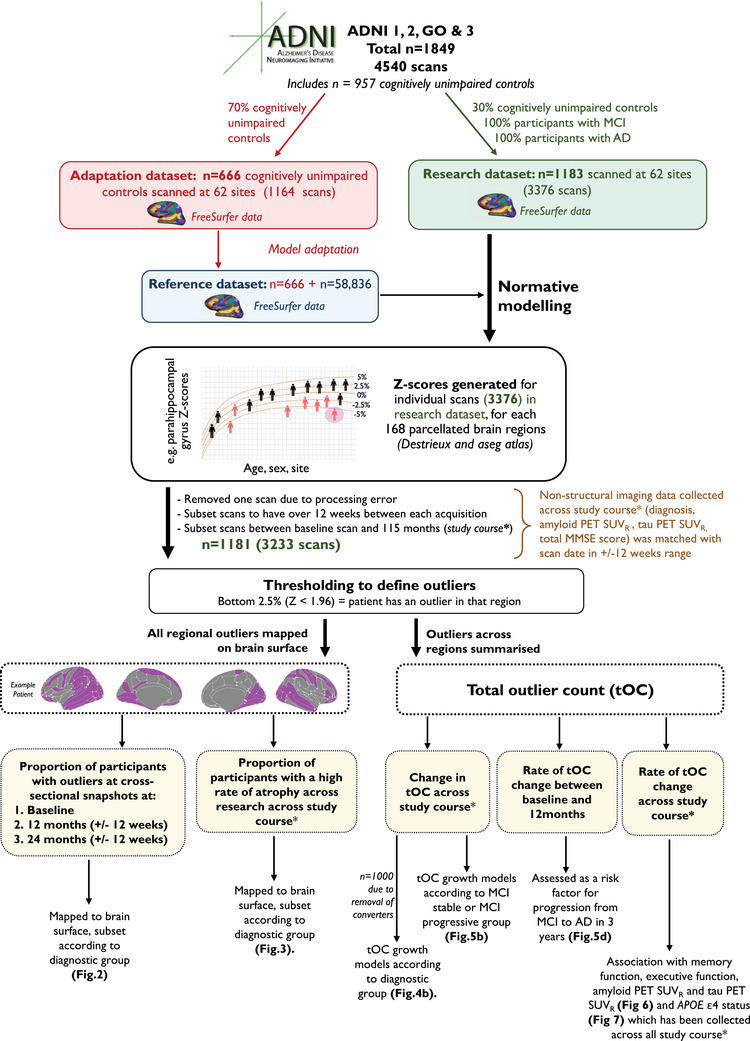
Flow diagram of methods pipeline, study inclusion criteria, and sample sizes. Diagnostic groups include cognitively unimpaired controls, participants with MCI, and patients with dementia. *Study course is between baseline scan (0 months) and 115 months/9.5 years. ADNI, Alzheimer's Disease Neuroimaging Initiative; *APOE*, apolipoprotein E; MCI, mild cognitive impairment; MMSE, Mini‐Mental State Examination; PET, positron emission tomography; SUVR, standardized uptake value ratio.

Participants were derived from two datasets (Figure 1): (1) a reference (training) dataset comprised of healthy people across the human lifespan, and (2) a research dataset that included people with AD or MCI in addition to age‐matched cognitively unimpaired controls. The reference dataset was made by combining T1‐weighted MRI scan data on healthy people from multiple publicly available sources,^19^ including Open Access Series of Imaging Studies (OASIS), Adolescent Brain Cognitive Development (ABCD) study, and UK Biobank (UKB), totaling 58,836 individuals from 82 sites. Data collection, data processing, and participant demographics of the reference dataset were described previously.^14^

The research data used in the preparation of this article were obtained from ADNI—http://adni.loni.usc.edu. Inclusion criteria were the availability of T1‐weighted MRI scans (acquired using 1.5T and 3T MRI scanners) from ADNI‐1, ‐GO, ‐2, and ‐3 (*n* = 1849, a total of 4540 scans). Participants had either a diagnosis of MCI or AD or were a cognitively unimpaired control, of which their diagnosis record date was matched ± 12 weeks after the scan date (see Table 1 for diagnosis sample sizes). Here participants with AD fulfilled the National Institute of Neurological and Communicative Disorders and Stroke–Alzheimer's Disease and Related Disorders Association criteria for probable AD,^20^ and patients were defined as having MCI or as cognitively unimpaired controls as described previously.^21^ Patients with MCI who developed AD during longitudinal follow‐up were classified as “MCI progressive” (*n* = 98) and the remainder as “MCI stable.” “MCI stable” excluded MCI regressors.

**RESEARCH IN CONTEXT**

1. **Systematic review**: The authors reviewed the literature using traditional (e.g., PubMed) sources. Alzheimer's disease (AD) atrophy is heterogeneous at the individual level throughout the disease course—neuroanatomical normative modeling is a quantitative technique to capture this. Relevant literature is cited.
2. **Interpretation**: Neuroanatomical normative modeling can generate personalized brain atrophy markers which can map changes over time in AD. We illustrate that neurodegeneration rates are heterogeneous over the disease course and reveal what cannot be seen using traditional group‐average statistics. This is consistent with previous AD and normative modeling research.
3. **Future directions**: Further validation of our personalized brain atrophy marker could be conducted in community‐based samples that comprise patients with both early‐ and late‐stage AD (to capture the full disease course). Future studies should assess if these individualized markers are sensitive to (1) capturing the deceleration of atrophy with disease‐modifying therapies and (2) being implemented as a decision‐making tool in clinical settings.

**TABLE 1:** Demographics and AD‐related characteristics of ADNI participants used in the study.

	Controls	MCI	AD	Total	Statistical differences
*n*	291	682	208	1181	–
Total of scans*	661	1999	573	3233	*–*
Median (IQR) of scans per participant	3 (2)	4 (3)	3 (2)	4 (3)	*–*
Follow‐up time in time series data (months) mean ± SD	41.5 ± 28.2	32.2 ± 25.5	23.8 ± 21.4	32.1 ± 25.9	*F*_(2, 767) _= 23.2, *p *= 1.5 × 10^‐10^
Sex (M:F)	121:170	381:301	106:102	608:573	*X* ^2^ = 16.68, *p =* 0.0002
Baseline age mean ± SD & range	73.9 ± 6.2 (56–93)	72.2 ± 7.8 (54–97)	74.1 ± 8.1 (55–90)	72.9 ± 7.6 (54–97)	*F*_(2, 1172) =_ 8.42, *p *= 0.0002
Baseline AC score (years)^†^ mean ± SD & range	–5.17 ± 5.83 (–15.3–9.8)	7.0 ± 4.0 (–7.0–14.6)	12.9 ± 2.1 (2.5–16.5)	4.3 ± 8.3 (–15.3–16.5)	*F*_(2, 724) _= 934.97, *p *= 2.2 × 10^‐16^
Baseline MMSE score mean ± SD total & range	29.07 ± 1.3 (22–30)	27.8 ± 1.9 (19–30)	22.6 ± 3.2 (7–30)	27.8 ± 1.9 (7–30)	*F*_(2, 1017) _= 561.3, *p *= 2.2 × 10^‐16^
Baseline ADNI memory mean ± SD total & range	1.2 ± 0.6 (–0.7–3.3)	0.3 ± 0.6 (–1.5–2.4)	–0.9 ± 0.6 (–2.9–0.4)	0.3 ± 0.9 (‐2.8–3.3)	*F*_(2, 1074) _= 590.5, *p *= 2.2 × 10^‐16^
Baseline ADNI executive function mean ± SD total & range	1.0 ± 0.8 (–1.2–2.9)	0.3 ± 0.8 (–2.3–2.9)	–0.9 ± 1.0 (–3.0–2.6)	0.3 ± 1.1 (–3.0–2.9)	*F*_(2, 1070) _= 248.9, *p *= 2.2 × 10^‐16^
Baseline amyloid PET SUVR mean ± SD total & range	1.1 ± 0.2 (0.9–2.7)	1.2 ± 0.2 (0.8–2.0)	1.4 ± 0.2 (0.9–1.8)	1.4 ± 0.2 (0.8–2.7)	*F*_(2, 630) _= 54.6, *p *= 2.2 × 10^‐16^
Baseline tau PET SUVR mean ± SD total & range	1.5 ± 0.2 (1.2–2.0)	1.6 ± 0.3 (1.2–2.6)	1.8 ± 0.4 (1.5–2.8)	1.6 ± 0.2 (1.2–2.8)	*F*_(2, 138) _= 7.9, *p *= 0.0005
*APOE* ε4 non‐carrier (percentage in group sample & sample size)	32.6% (*n* = 194)	55.6% (*n* = 332)	11.7% (*n* = 70)	53% (*n* = 596)	**X ^2^ = 107.5, *p *= 2.2 × 10^‐16^

*Note*: Key: **X^2^ of *APOE* ε4 status.

Abbreviations: AD, Alzheimer's disease; ADNI, Alzheimer's Disease Neuroimaging Initiative; *APOE*, apolipoprotein E; IQR, interquartile range; MCI, mild cognitive impairment; PET, positron emission tomography; SD, standard deviation; SUVR, standardized uptake value ratio.

^†^ Disease stage (AC) score represents predicted years since amyloid PET positivity.

Furthermore,[Fig alz14174-fig-0001] additional variables were obtained from the research dataset (ADNI) and linked to each MRI assessment, assuming these additional data were acquired within 12 weeks of the scan. This included cognitive data: memory using ADNI memory (MEM) or executive function using ADNI executive function (EF),^22^ and the Mini‐Mental State Examination (MMSE) total score^23^; amyloid and tau markers: for amyloid, florbetapir, a summary positron emission tomography (PET) standardized uptake value ratio (SUVR),^24^ and tau, a PET summary SUVR flortaucipir^25^; and genetic markers: *APOE* ε4 status (determined by either being *APOE* ε4 homozygous, *APOE* ε4 heterozygous, or *APOE* ε4 non‐carrier), and previously generated polygenic risk score (PRS).^26^

In addition, we included a numerical index designed to reflect the AD stage, the so‐called amyloid‐cognition score (AC score). AC scores were calculated using latent time disease progression modeling of longitudinal amyloid PET SUVR and cognitive clinical scores, using methods previously detailed.^27^, ^28^ AC scores have been proposed as a natural timescale to compare biomarker trajectories and have previously been shown to stage patients along the AD continuum more precisely than conventional measures (e.g., early or late MCI).^27,28^

### 2.2 MRI acquisition

For ADNI, T1‐weighted images were acquired at 62 study sites using 1.5T or 3T MRI scanner visits across ADNI‐1, ‐GO, ‐2, ‐3. Detailed MRI protocols for T1‐weighted sequences are available online (http://adni.loni.usc.edu/methods/documents/mri‐protocols/). The quality of raw scans was evaluated at the Mayo Clinic for technical problems and significant motion artifacts and clinical abnormalities.^29^

### 2.3 Estimation of cortical thickness and subcortical volumes

T1‐weighted scans from both the reference and the research dataset were processed using FreeSurfer *recon‐all* cross‐sectional, to extract the cortical thickness of 148 cortical regions and gray matter tissue volume of 20 subcortical volumes from the Destrieux parcellations.^30^, ^31^ For the reference dataset FreeSurfer version 6 was used; for ADNI, FreeSurfer versions 5 or 6 were used. Quality control of FreeSurfer processing for the reference dataset relied on both manual and automated filtering, as described previously.^14^ For the research dataset, quality control was based on a visual review of each cortical region performed at University of California San Francisco (https://adni.bitbucket.io/reference/docs/UCSFFSX51/UCSF%20FreeSurfer%20Methods%20and%20QC_OFFICIAL.pdf).

### 2.4 Neuroanatomical normative modeling

A non‐Gaussian Bayesian regression model was implemented, which accounts for the non‐Gaussian distributions of the cortical thickness and subcortical volume data and adjusts for unwanted noise from scanning acquisition across multiple sites.^32^ This model was trained on 58,836 scans from datasets across 82 sites to generate normative models per region using the covariates age, sex, and site, as previously described.^14^, ^32^ Next, these estimates were calibrated to our specific test data (i.e., ADNI), using an adapted transfer learning approach.^19^ The distribution parameters of the reference normative model were calibrated to our ADNI dataset using 70% of cognitively unimpaired controls per ADNI site, with the aim of reducing site effects and software version effects (i.e., FreeSurfer v5 or v6). The controls for calibration were randomly selected with stratification to ensure all sites and sexes were present in the adaptation set. The data from the remaining 30% of cognitively unimpaired controls, plus participants with MCI and patients with AD, were then compared to these normative models, generating *z* scores per region for each scan. A summary of this pipeline and a breakdown of the respective sample sizes included are detailed in Figure 1. The final sample used in *z* score analysis is *n* = 1181, which included 3233 scans (Figure 1 and Table 1). The modeling steps and models trained on the reference dataset are openly available: https://github.com/predictive‐clinical‐neuroscience/braincharts.

#### 2.4.1 Individualized brain markers

Extreme deviations from the norm, or “outliers,” with lower cortical thickness and subcortical volumes were identified for each region, defined as *Z* < –1.96. Here ventricular *z* scores were inverted to reflect volumetric expansion. The number of outliers was summed across all 168 regions to give a total outlier count (tOC) for each participant. Brain surface mapping was conducted using the Destrieux (148 cortical regions) and aseg (20 subcortical regions) atlas via the R package ggseg. All statistical analyses were implemented in R version 3.6.2.

### 2.5 Disease course analysis

#### 2.5.1 Outliers at three cross‐sectional snapshots over a 24‐month period

For these analyses, longitudinal data were subset into three time points representing a cross‐sectional snapshot measure of baseline, month 12 (in a range of ± 12 weeks), and month 24 (in a range of ± 12 weeks). Brain outlier maps for each diagnostic group were mapped across the three time points. This enabled visualization of the extent to which patterns of outlier regions overlap or are distinct in each of these three time points.

#### 2.5.2 Rate of change in tOC and regional *z* scores

To assess longitudinal atrophy, we took two related approaches. First, we calculated the rate of change in tOC as the difference in baseline and final tOC (up to 115 months). A linear model was used to test for differences in the rate of change in tOC between diagnostic groups while adjusting for age, sex, and predicted AD stage (AC score, see Section 2.1).^28^, ^33^ Second, we calculated the rate of change in *z* score per region. Here, we calculated the difference between the baseline and final *z* score, and then we defined a new “normative model” based on the distribution of rates of change in scans of cognitively unimpaired controls reserved for analysis (661 scans). “Rate of *z* score change outliers” was then defined if a rate of change was more than two standard deviations away from the mean in the ADNI controls (which was *Z* = –0.0009). Then, the neuroanatomical patterns of the “rate of *z* score change outliers” were mapped onto brain surfaces for visualization purposes. As a final step, to provide more detail, we focused on the region of the highest rate of change (in this case, the left hippocampus), and compared patients with AD who had a “rate of *z* score change outliers” to those that were not, based on their total MMSE score and their AD stage (AC score), age, sex, and *APOE* ε4 status.

#### 2.5.3 Growth models

Growth models were used to understand individual trajectories of tOC over time using the lme4 package in R. Here, separate unconditional linear mixed models were generated for each of the three diagnostic groups; tOC is considered a dependent variable and time as the independent variable. The “control to MCI” and “MCI to AD” converters were removed from this model to ensure the diagnostic group data is distinct across each individual timeline (total *n* = 181 removed; Figure 1). Individual participants were considered variables acting as a random intercept (which allows subjects to vary randomly in terms of their intercept) and therefore models each participant's tOC linearly with time, taking into account how individual participants vary in the slopes and intercepts of this relationship.^34^

#### 2.5.4 MCI to AD progression analysis

Data were subset for participants who had MCI diagnosis at baseline and also had follow‐up data for 3 years (36 months) since their baseline visit (*n* = 365). Using this subset, we ran a survival analysis using Cox proportional hazards regression to assess whether the tOC difference between baseline and 12 months was related to the risk of progression from MCI to AD in 36 months. The tOC change was thresholded according to the median tOC change between baseline and 12 months (median = 3) to signify a low or high rate of change in the first year and a Kaplan–Meier plot was used to illustrate the progression from MCI to AD. We also compared the first‐year change in tOC between MCI stable and MCI progressive groups. Furthermore, we added baseline tOC to our Cox proportional hazards regression model (alongside tOC change in the first year) to explore the relationship of baseline tOC to risk of progression.

### 2.6 Relationship to other disease markers

#### 2.6.1 AC score

The AC score, representing predicted years since amyloid PET positivity, was used as an approach for staging AD (see Section 2.1). This continuous measure was used as a covariate to adjust for disease stage when exploring how tOC relates to cognitive, amyloid, and tau AD disease markers.

#### 2.6.2 Cognitive markers

Linear regression adjusting for age, sex, and years of education examined the relationship between tOC rate of change and cognitive composite scores (memory using ADNI MEM or executive function using ADNI EF).^34^ We then assessed the interaction between the diagnostic group and cognitive composite score. Total MMSE scores were only used when comparing regional rates of change (Figure 1).

#### 2.6.3 Amyloid and tau markers

Florbetapir and flortaucipir SUVR were used to index cerebral amyloid and tau deposition, respectively. A linear regression adjusting for age and sex examined the relationship between the rate of change in tOC and amyloid and tau PET markers. We assessed the interaction between the diagnostic group and amyloid and tau markers in a subsequent regression.

#### 2.6.4 Genetic markers

*APOE* ε4 status was determined by either being *APOE* ε4 homozygous, *APOE* ε4 heterozygous, or *APOE* ε4 non‐carrier. Group differences in the rate of change in tOC were assessed as a linear regression, adjusting for age and sex. The relationship between PRS and the rate of change in tOC was examined using linear regression, adjusting for age, sex, and *APOE* ε4 status. We assessed the interaction between the diagnostic group and PRS in a subsequent regression.

## 3 RESULTS

### 3.1 Participants

The final research dataset amounted to a total of 1181 participants with a total of 3233 scans at least 12 weeks apart between scanning visits and had a maximum interval between baseline and final visit of 9.5 years (115 months; Figure 1 and Table 1).

### 3.2 The change in neuroanatomical outliers between baseline and 24 months

**FIGURE 2:**
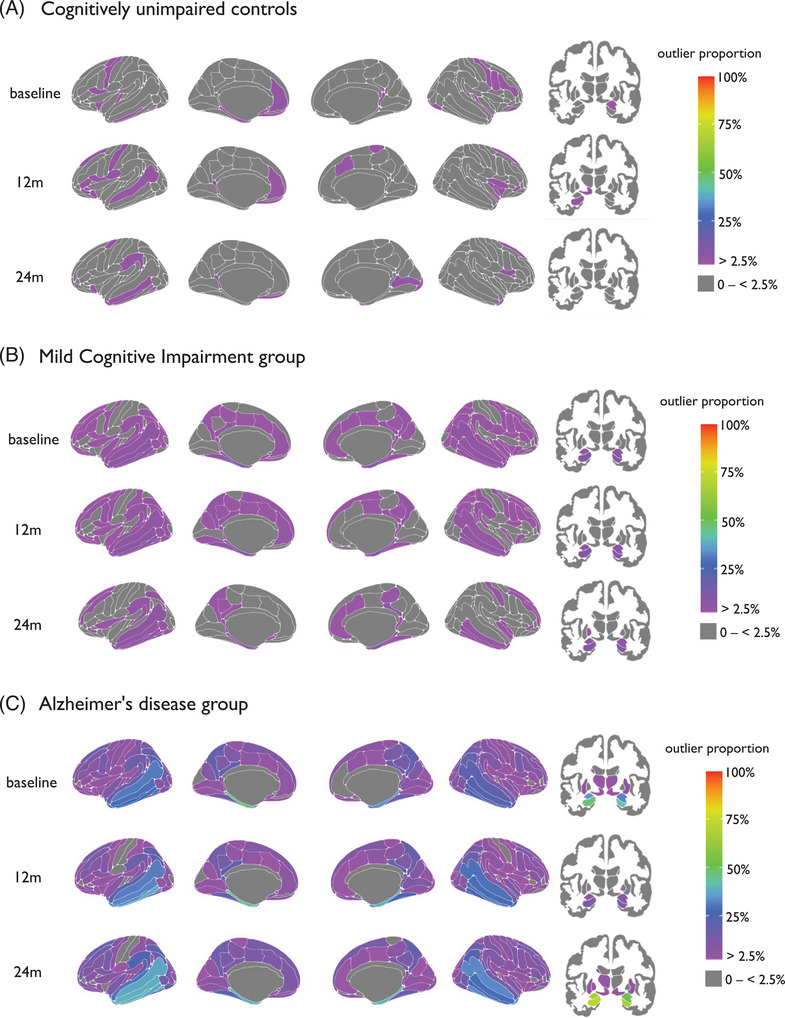
Mapped are the percentage of outliers present in (A) cognitively unimpaired controls, (B) mild cognitive impairment, and (C) Alzheimer's disease at baseline, 12 months, and 24 months, for cortical (left) and subcortical (right) areas. The color bar reflects the outlier proportion from 2.5% to 100% (thresholding of *z* scores). Zero percent (gray) represents that no participants have outliers in those respective regions.

Patterns of cortical thickness and subcortical volume outliers differed between AD, MCI, and control groups and varied over time (Figure 2). The proportion of outliers in each group was mapped cross‐sectionally at baseline, 12 months, and 24 months. In patients with AD, the region with the highest proportion of the group having outliers was consistently the left hippocampus, with 47% at baseline, 60% at 12 months, and 72% at 24 months. Generally, the number of regions that contained at least one participant with an outlier remained stable; at baseline, there were 134 cortical regions and 13 subcortical regions with outliers, 128 and 12 at 12 months and 131 and 11 at 24 months, respectively.

### 3.3 Higher rate of atrophy in patients with AD

**FIGURE 3:**
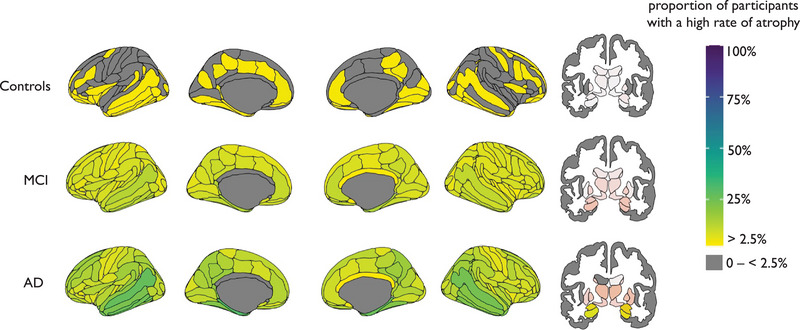
Mapped is the proportion of participants with a high rate of atrophy in cortical (left) and subcortical (right) areas. AD, Alzheimer's disease; MCI, mild cognitive impairment.

Overall, patients with AD showed a greater number of “rate of *z* score change” outliers (see Section 2.5.2), compared to people with MCI or controls. The region with the highest proportion of rate of *z* score change outliers was the left hippocampus at 53%; therefore, 47% of patients with AD patients do not follow this trend (Figure 3). Comparing patients with AD that did versus did not have rate of *z* score change outliers in the left hippocampus, there were no statistical differences in age, total MMSE score, and AC score at baseline (*p *> 0.05), and no statistical differences in *APOE* ε4 carrier status or sex (*X^2^ P *> 0.05). In the 47% of patients who did not show rate of *z* score change outliers in the left hippocampus, the regions with the highest proportion of patients with outliers were the left amygdala (29%), the right amygdala (28%), and left and right lateral ventricles (both 11%).

### 3.4 tOC increases with time in AD and in individuals with MCI who progress to AD

**FIGURE 4:**
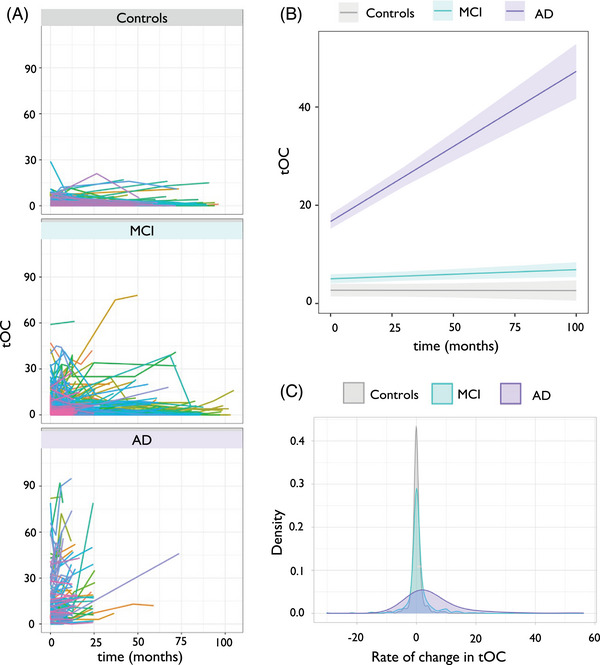
Change in tOC according to diagnostic group. A, Spaghetti plot of tOC according to diagnostic group. Each colored line represents an individual participant's trajectory of tOC scores over the scanning period. B, Linear growth model for each diagnostic group. C, The density spread of the rate of change in tOC for each diagnostic group. AD, Alzheimer's disease; MCI, mild cognitive impairment; tOC, total outlier count.

Increased tOC over time (i.e., accumulation of outliers) was observed in the AD group (*β* = 0.38, *p *= 9.8 × 10^‐13^) and in the MCI group (*β *= 0.001, *p *= 0.004) but not in controls (*β* = –0.001, *p *= 0.81; Figure 4A,B). Linear regression revealed that there was a significant increase in the rate of tOC change over time (Figure 4C). This differed between groups when adjusting for baseline AC score, baseline age, and sex (*F*_(5, 490) _= 12.99, *p *= 6.9 × 10^‐12^). Interestingly, when including a quadratic term for time (i.e., time^2^) to model the non‐linear effects of time, this was significant (*β *= –31.0, *p *= 0.0005). When assessing the interaction between group and time^2^ we saw that this effect was stronger in AD patients (*β *= –35.5, *p *= 0.06) than in people with MCI (*β *= –84.07 *p *= 0.0005). Pairwise group comparisons (Tukey post hoc tests) of the different group trajectories over time were significant (*p *≤ 0.001) for AD versus controls and AD versus MCI, but not controls versus MCI (*p *= 0.518). The rate of change in tOC was highest in the AD group (mean = 5.26, standard deviation [SD] = 9.85), intermediate in the MCI group (mean = 0.59, SD = 4.57), and lowest in controls (mean = –0.01, SD = 1.90). AC score was significantly associated with the rate of change in tOC across the whole sample when adjusting for age and sex (*β* = 0.24, *p *= 7.5 × 10^‐09^).

**FIGURE 5:**
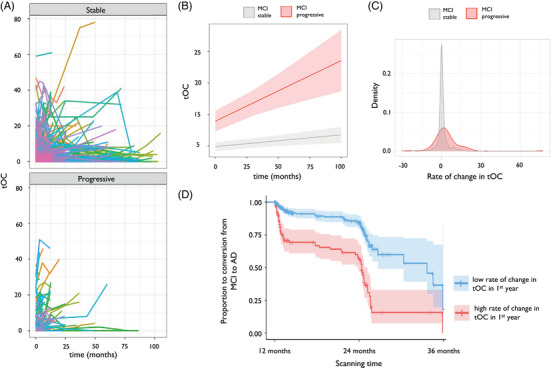
Change in tOC according to disease conversion status. A, Spaghetti plot of tOC in either MCI stable or MCI progressive. Each colored line represents an individual change in tOC over the scanning period. B, Linear growth model for MCI stable or MCI progressive. C, The density spread of the rate of change in tOC in people with MCI. D, Kaplan–Meier plot of MCI progression to AD between 12 and 36 months: the two lines represent a median split of tOC, with < 3 classed as low tOC (blue), and ≥ 3 classed as high tOC (red). Crosses indicate censoring points (i.e., time from baseline at last diagnosis assessment). Filled colors represent the 95% confidence intervals. AD, Alzheimer's disease; MCI, mild cognitive impairment; tOC, total outlier count.

Growth models showed that an increase in tOC over time was observed in both the MCI progressive group (*β *= 0.10, *p *= 7.4 × 10^‐05^, which equates to 1 additional tOC every 10 months) and the MCI stable group (*β *= 0.017, *p *= 0.004; Figure 5A,B). Linear regression showed that the MCI progressive group had a significantly higher rate of tOC change over time (mean = 3.97, SD = 11.27) compared to the MCI stable group (mean = 0.53, SD = 4.61), when adjusting for age, sex, and AC score (*F*_(4, 214) _= 2.622, *p *= 0.03; Figure 5C). There was a significant difference in the rate of change in the first 12 months between the MCI stable and progressive groups (*β *= 4.47, *p *= 3.1 × 10^‐15^). Survival analysis indicated that for every three outliers increase in tOC in the first 12 months, the risk of progression from MCI to AD between 12 months and 36 months (i.e., in the following 2 years) increased by 30.2% (hazard ratio [HR] = 1.09, 95% confidence interval [CI]: [1.06, 1.12], *p *= 1.4 × 10^‐14^; Figure 5D). When adding baseline tOC as another predictor to our Cox proportional hazards regression model (alongside tOC change in the first year), survival analysis indicated that for every three outliers increase in baseline tOC, the risk of progression from MCI to AD between 12 months and 36 months increased by 9.7% (HR = 1.03, 95% CI: [1.01, 1.05], *p *= 0.003).

### 3.5 Rate of change in tOC correlates with cognitive, amyloid, and tau markers

**FIGURE 6:**
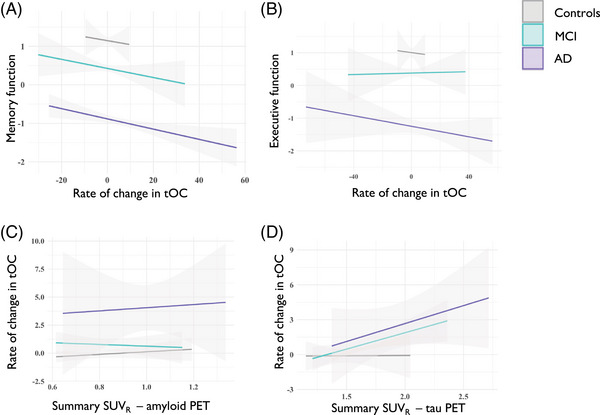
The relationship between cognitive function and cerebrospinal fluid markers with the rate of change in tOC. Fitted lines are from a linear regression model per diagnostic group for (A) memory function, (B) executive function, (C) summary SUVR amyloid PET, (D) summary SUVR tau PET. AD, Alzheimer's disease; MCI, mild cognitive impairment; PET, positron emission tomography; SUVR, standardized uptake value ratio; tOC, total outlier count.

The rate of change in tOC across the whole sample was significantly associated with poorer memory performance (*β* = –1.99, *p *= 2.0 × 10^‐16^), and executive function (*β* = ‐1.77, *p* = 2.0 × 10^‐16^) in separate linear regression models, controlling for age and sex (Figure 6A,B). Here interactions between the diagnostic group and memory (*F*_(2, 730 _= 4.95, *p *= 0.007) and diagnostic group and executive function (*F*_(2, 722) _= 4.44, *p *= 0.012) were both significant and were driven by patients with AD (memory [*β* = –2.52, *p =* 0.011], executive function *β* = –1.73 *p =* 0.013]). The rate of change in tOC across the whole sample was significantly associated with an increase in amyloid PET summary SUVR (*β *= 0.006, *p *= 0.001) and an increase in tau PET summary SUVR (*β *= 0.03, *p *= 0.0001) when adjusting for age and sex (Figure 6C,6D). Interactions between group and SUVR were not significant (*p *> 0.05).

### 3.6 Rate of change in tOC is associated with *APOE* ε4 status

**FIGURE 7:**
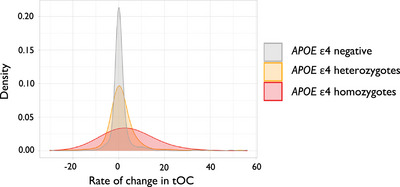
The density spread of the rate of change in tOC according to *APOE ε*4 status. *APOE*, apolipoprotein E; tOC, total outlier count.

*APOE* ε4 status showed differences in the rate of change in tOC, in an analysis of variance adjusting for age and sex (*F*_(2, 755) _= 10.06, *p *= 4.8 × 10^‐05^), which was not influenced by diagnostic group x *APOE* ε4 status interaction (*p *> 0.05). This association was driven by higher accumulation of outliers in *APOE* ε4 homozygotes (*β *= 2.33, *p *= 0.003) and *APOE* ε4 heterozygotes (*β *= 5.77, *p *= 0.018) compared to *APOE* ε4 negative participants (*β *= –0.65, *p *= 0.185; Figure 7). Linear regression showed no significant association between PRS and rate of change of tOC when adjusting for age, sex, and *APOE* ε4 status (F_(1, 707) _= 1.41, *p *= 0.23), which was not influenced by diagnostic group x PRS interaction (*p *> 0.05).

## 4 DISCUSSION

In this study we used neuroanatomical normative modeling to capture individual patient trajectories of brain structure changes during MCI and after AD diagnosis. Our normative modeling approach generates patient‐specific regional outlier maps, and summary outlier scores (tOC), which we analyzed using serial MRI data acquired up to 9.5 years (mean follow‐up time 2.4 years) as part of ADNI. Our findings illustrate that AD affects patients in a non‐uniform way as the disease progresses.

A key advantage of neuroanatomical normative modeling is that it provides region‐level information, with separate normative models per brain region. We observed that patterns of outliers vary over time in the AD group; for instance, the percentage of patients with AD that had outliers in the hippocampus increased from 47% to 72% in 24 months, suggesting the presence of atrophy in the hippocampus is more heterogenous in earlier stages of the disease and becomes more common as the disease progresses (Figure 2).

This is consistent with our finding that the rate of *z* score change was highest in the left hippocampus, with 53% of the AD group having outliers (i.e., greater than expected changes in *z* score) in this area (Figure 3). Hippocampal atrophy is seen as characteristic of AD and is included in AD diagnostic criteria, as well as being used in clinical trials.^35^ However, our results show that in the ADNI AD sample, 47% of patients do not have greater‐than‐expected left hippocampal volume changes. These results highlight the individual differences between patients with AD and emphasize the limitation of group‐average statistical designs, which would overlook this within‐group variability. Moreover, we found that age, sex, total MMSE score and AD stage (AC score), and *APOE* ε4 status were not associated with either *having* or *not having* a marked rate of *z* score change outliers (i.e., elevated atrophy), which may provide further evidence for a hippocampal‐sparing subtype.^36^, ^37^, ^38^, ^39^

Our results are consistent with previous work on neuroanatomical variation in dementia. When using a related neuroanatomical normative modeling technique (hierarchical Bayesian regression^40^), we previously found that patients with AD had a higher tOC and large interindividual differences in regional outliers at baseline, compared to people with MCI and cognitively unimpaired controls.^17^, ^18^ Here, our longitudinal data showed an increase in tOC in patients with AD. Interestingly, the rate of change in tOC in the AD group increased in a non‐linear way, suggesting an accelerating accumulation of brain structural outliers over the study period. Therefore, tOC could offer some utility in tracking neurodegeneration across the disease course in individuals with AD (Figure 4).

Alongside capturing accumulating atrophy in AD, people with MCI who later progress to AD have increasing rates of tOC (Figure 5A–C). This is consistent with our previous research that showed an increase of 10 outliers (i.e., +10 tOC) confers a 31.4% chance of clinical progression within 3 years.^17^ Here, we also found that the rate of change in tOC over 1 year (i.e., the difference in tOC between baseline and 12 months), was associated with progression to dementia in the subsequent 2 years, with an increase of three outliers in this first year giving 30.2% increased risk of clinical progression between 12 months and 36 months (Figure 5D). This raises the possibility that normative modeling approaches to serial scans in patients with MCI could have utility in detecting those that will progress to AD dementia and that annual scans for people with MCI to monitor brain health could benefit the clinical decision‐making process (e.g., for early AD detection). Interestingly, some individuals showed negative rates of tOC, thus having fewer outliers over time. This includes 18% of cognitively unimpaired people, 12.7% of people with MCI, and 10.8% of AD patients, though for the vast majority of these participants the decrease was < 5 regions, thus could be caused by noise in the MRI scans leading to minor changes to the FreeSurfer output.

We observed that the rate of change in tOC was associated with amyloid and tau PET SUVR (Figure 6C,6D), in line with previous associations of amyloid and tau with neuroanatomical changes in AD,^41^, ^42^, ^43^, ^44^ although it is important to note that the amyloid/tau/neurodegeneration (ATN) interplay is likely to differ from individual to individual.^45^, ^46^ We also assessed how genetic factors could relate to tOC change over time. We found that an increased tOC is associated with *APOE* ε4 homozygosity and heterozygosity (Figure 7), consistent with other structural imaging markers findings, and is likely to reflect amyloid load.^26^, ^47^, ^48^ Likewise, our results indicate that memory and executive function are associated with the accelerated accumulation of outliers (Figure 6A,B).^49^, ^50^ This highlights that participants with outliers are more likely to have (1) a clinical diagnosis of AD, (2) to be AD biomarker positive, and (3) to have cognitive features consistent with AD, reflecting alignment with current clinical frameworks.^51^, ^52^

Indeed, accelerated brain atrophy is a widely accepted marker of AD,^4^, ^53^ thus rate of change in brain structure outliers (i.e., tOC) may offer better clinical utility than using raw brain volumes/thicknesses. Therefore, in a clinical setting, a patient's tOC could be derived from serial MRI scans to track brain changes over time. Similar brain structure measures derived from normative modeling have already been considered for clinical translation; the Quantitative Neuroradiology Initiative (QNI) provides a framework to contextualize a dementia patient's brain health and provide a personalized score to support clinical decision making.^54^, ^55^ Building on this idea, our application of neuroanatomical normative modeling offers regional information on brain health (mapped outlier scores), and improved neuroanatomical normative model estimates by using a large reference cohort.^56^

One setback with translating computational statistical designs in clinical settings is the technical barriers to application and limits to data sharing. However, our neuroanatomical normative modeling approach does not require access to raw scans, as the end user only requires a pre‐trained reference model, which contains no identifiable data. Scripts to generate individual *z* scores, tOC, and outlier maps are openly available.^57^

Neuroanatomical normative modeling also has the potential to aid in trials of AD therapeutics. For example, it could be used to stratify people for trial enrolment based on the extent (tOC) and spatial distribution of their brain atrophy, to identify subgroups based on different atrophy patterns or as a personalized outcome measure, for which the impact of the treatment using unique brain “fingerprints” can be quantified, increasing power and sensitivity to subtle changes over time.^9^ Moreover, with further validation, it could warrant a run‐in period of 12 months in clinical trials, during which an enrolled participant is stratified by their progression risk, based on the accumulation of neuroanatomical outliers over the previous year.

Yet, prior to clinical and drug trial implementation, additional diversification of datasets is needed. Although our reference dataset is large, it is over‐representative of European ancestry due to the datasets predominantly from research studies (which do not match either regional or global population demographics).^58^, ^59^ Though ADNI participants are mostly of European ancestry,^60^ caution should be made when transferring the model to diverse datasets, or participants from underrepresented demographics.^61^ Moreover, ADNI participants are more likely to be in the early to intermediate stages of AD, and less likely to have comorbidities.^21^, ^61^, ^62^, ^63^ Future work will require the reference dataset and research/patient datasets to include participants from non‐research studies (e.g., participants from memory clinics,^18^ which are more likely to include later‐stage patients with more comorbidities), and different social–economic backgrounds and ethnicities to reduce bias and mitigate health‐care inequalities.^64^

Further optimization of neuroanatomical normative modeling is possible. Although scanner effects and the non‐Gaussian distribution of the neuroimaging phenotypes were accounted for, some within‐subject noise remains in longitudinal data, which may be contributing to the range in tOC change (Figure 5C and Figure 6C). To assess this, it will be useful to understand test–retest reliability by calculating the difference in scans that have been acquired in close succession (< 1 week).^65^ Also, the within‐subject variability may have been better modeled by implementing the longitudinal FreeSurfer processing pipeline.^66^ ADNI data were processed with a variety of FreeSurfer versions (5 & 6). While impractical to unify the processing retrospectively, these inconsistencies may add noise to the normative models from potential differences in cortical thickness and subcortical volume estimates,^67^, ^68^ although there is evidence for consistencies of these estimates between some FreeSurfer versions.^69^ Furthermore, our model treats brain regions independently, yet it is likely that regional *z* scores are intercorrelated, particularly between neighboring or bilateral regions. Solutions to this could consider the spatial extent of affected voxels and the magnitude in those voxels,^70^ and apply normative models that use brain connectivity data, which have shown recent promise.^58^

To conclude, we show that brain structural outliers across MCI and AD differ at the individual level and that this can be visualized over time by using outlier maps and quantified by the tOC generated by neuroanatomical normative modeling. Our study further supports the potential utility of tOC and brain outlier maps as personalized markers for patients with AD and to assess the risk of disease progression in people with MCI. The next steps are to diversify the training and research/patient data used, and further validate these markers for future translation into clinical settings and in clinical trial design.

## CONFLICT OF INTEREST STATEMENT

L.L.R. is an employee of Eli Lilly and Company. All other authors report no disclosures relevant to the manuscript. Author disclosures are available in the supporting information.

## CONSENT STATEMENT

All data were approved by each cohort's respective institutional review board and informed written consent was obtained from all participants.

## Supporting information

Supporting Information
